
JOURNAL INFORMATION
==============================
NLM Title Abbreviation: Rev Soc Bras Med Trop
Journal ID: Rev Soc Bras Med Trop
NLM Journal ID: 7507456
Journal ID: rsbmt

Revista da Sociedade Brasileira de Medicina Tropical

ISSN: 0037-8682
EISSN: 1678-9849
Publisher: Brazilian Society of Tropical Medicine

ARTICLE INFORMATION
==============================
PMCID: PMC9131776
PMID: 35613223
DOI: 10.1590/0037-8682-0233-2022
Article ID: 01000
Article version: 1
Subjects: Technical Report

Are we near the end of the pandemic?

http://orcid.org/0000-0003-4826-3355 Maciel Ethel Leonor 1 2
http://orcid.org/0000-0002-9662-1930 Oliveira Wanderson Kleber 3
http://orcid.org/0000-0002-3346-3509 Siqueira Priscila Carminati 2
http://orcid.org/0000-0002-6665-6825 Croda Julio 1 4 5 6
1 Rede Brasileira de Pesquisa em Tuberculose, Rio de Janeiro, RJ, Brasil.
2 Universidade Federal do Espírito Santo, Programa de Pós-Graduação em Saúde Coletiva, Vitória, ES, Brasil.
3 Supremo Tribunal Federal, Brasília, DF, Brasil.
4 Fundação Oswaldo Cruz, Campo Grande, MS, Brasil.
5 Universidade Federal de Mato Grosso do Sul, Faculdade de Medicina, Campo Grande, MS, Brasil.
6 Yale School of Public Health, Department of Epidemiology of Microbial Diseases, New Haven, CT, USA.
Corresponding author: Ethel Leonor Maciel. e-mail: ethel.maciel@gmail.com Authors’ contribution: ELM, WKO, PCS, JC all authors contribute to the conception, design of the study and interpretation of data, as well as the final approval of the version to be submitted.

Conflict of Interest: The authors declare that there is no conflict of interest.

Electronic publication date: 2022 May 20
Publication date: 2022
Volume: 55
Electronic Location ID: e0233-2022
Received 2022 Apr 21; Accepted 2022 May 3
License: Este é um artigo publicado em acesso aberto sob uma licença Creative Commons
License URL: https://creativecommons.org/licenses/by/4.0/

Figure count: 2
Table count: 0
Equation count: 0
Reference count: 17

==============================

***Ethel Leonor Maciel[1],[2], Wanderson Kleber Oliveira[3], Priscila Carminati Siqueira[2] and Julio Croda[1],[4],[5],[6]***

To answer this question, we need to understand which concepts are being discussed. Are we talking about epidemiological or operational concepts? Additionally, what practical implications are applicable to each concept? In the epidemiological dimension, there are outbreaks, epidemics, pandemics, and endemics. In an endemic, the presence of the disease occurs within an expected pattern for the time of year within a given geographical area or population group. When the number of new cases is high, it is described as hyperendemic. Thus, the incidence of an endemic disease is relatively constant, and seasonal variations can occur in the expected behavior of the disease. In the definition of an epidemic, the occurrence of the disease is above the historical average (or median) of its occurrence. The disease causing an epidemic usually has a sudden seasonal onset and spreads for a certain period in a given geographical area, often affecting a large number of people[1-3]. When an epidemic affects several countries on different continents, it is called a pandemic, as is the case with Coronavirus disease 2019 (COVID-19)[4].

In the operational scope, according to the National Health Surveillance Policy (*Política Nacional de Vigilância em Saúde*), a Public Health Emergency is a situation that demands the urgent use of measures to control and contain risks to prevent damage and harm to public health[5]. This concept was derived from the International Health Regulations that, from 2005 onwards, started to define extraordinary events, related to diseases or threatened to cause diseases, that constituted risks to the public health of other countries, due to international spread and requiring a coordinated international response[5].For this reason, on January 30, 2020, the World Health Organization (WHO) declared a Public Health Emergency of International Importance due to COVID-19[6]. At the time, it was not a pandemic but later it was justified by the dispersion and magnitude of the events. According to the WHO, a pandemic is characterized when the disease is recorded in at least three of the six administrative regions (continents).

In Brazil, Decree No. 7616 of November 17, 2011, supports the declaration of a public health emergency. For this, the administrative act is published in the Official Gazette specifying a situation that can be epidemiological, due to disasters, or due to a lack of assistance to the population[7].

In the situation of the pandemic of COVID-19, as it was an emerging disease, we did not have previous parameters of the disease; also the definition of consensual parameters for the finalization of the pandemic decree had not yet been defined by the international health authorities. For infectious diseases, we used indicators such as the number of cases per 100 000 population and the number of deaths per 10 000 population to define patterns of endemicity and disease control[8].

On March 11, 2020, the date referring to the epidemiological week (EW) 11/2020, the World Health Organization declared that the outbreak of COVID-19 was a public health emergency of international concern, thus announcing the pandemic[4]. At the time of EW 11/2020, 138 cases and no deaths had been reported in Brazil., Since then, the first "peak" of the pandemic occurred in EW 30/2020, with 321,006 confirmed cases. Since then, the period with the lowest record of confirmed cases occurred between EW 50/2021 and 51/2021, with 24,247 and 22,181 confirmed cases, respectively. From this time, there had been a significant increase in the number of cases until EW 04/2022, with the highest incidence in the historical series. A total of 1, 324, 589 confirmed cases were recorded in a single epidemiological week. However, after 10 weeks, there was a sharp reduction, decreasing to 151,194 cases in EW 14/2022[8].

Despite being a lower number, even considering better vaccination coverage and manageable pressure from the service network, this level is not yet safe to abolish prevention and control measures; configuring the interepidemic and non-endemic phases, we may move on to an "endemic" process in the future, or we may even observe new "peaks" as has occurred in European countries.

Currently, we are in the pandemic transition phase. Risk assessments are initially made when viruses with pandemic potential are identified, and data are updated according to the characteristics of the viruses, as well as epidemiological and clinical data. The risk-based approach to the pandemic phases is represented in **Figure 1A-B** as a continuum. The transition phase is characterized by a decrease in overall risk. After the transition phase, the inter-pandemic phase commences[9].

The transition to endemicity would mean our ability to "live with the virus," without restrictions and additional protective measures, due to the vaccine. Eventually, the use of individual preventive measures for certain situations, risk groups, epidemic periods may be required, and if there is need, we may have to institute additional preventive measures, since the virus and the disease will remain amongst us.

Endemicity in COVID-19 will, therefore, depend on our immunity to the virus, as most people have already been exposed to it, either by vaccine or in conjunction with prior infection, which we call hybrid immunity. A herd immunity threshold is uncertain, because of the impact of new variants and duration of protection. We are not yet sure whether we will need new vaccines

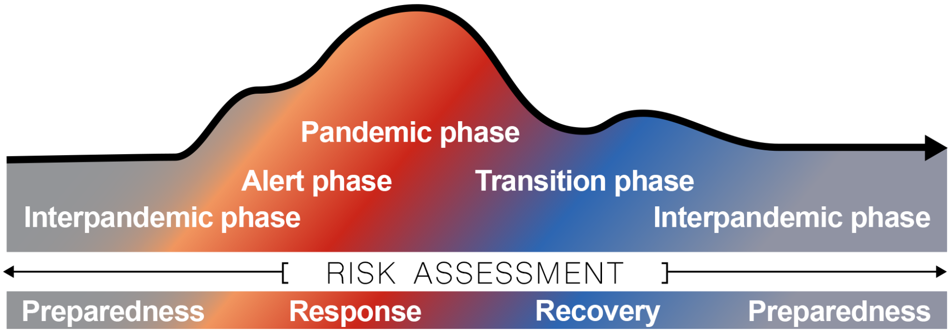

**FIGURE 1A:** The continuum of the phases of the pandemic: Influenza.
**Source:** WHO, 2017. Pandemic influenza risk management: A WHO guide to inform and harmonize national and international pandemic preparedness and response.

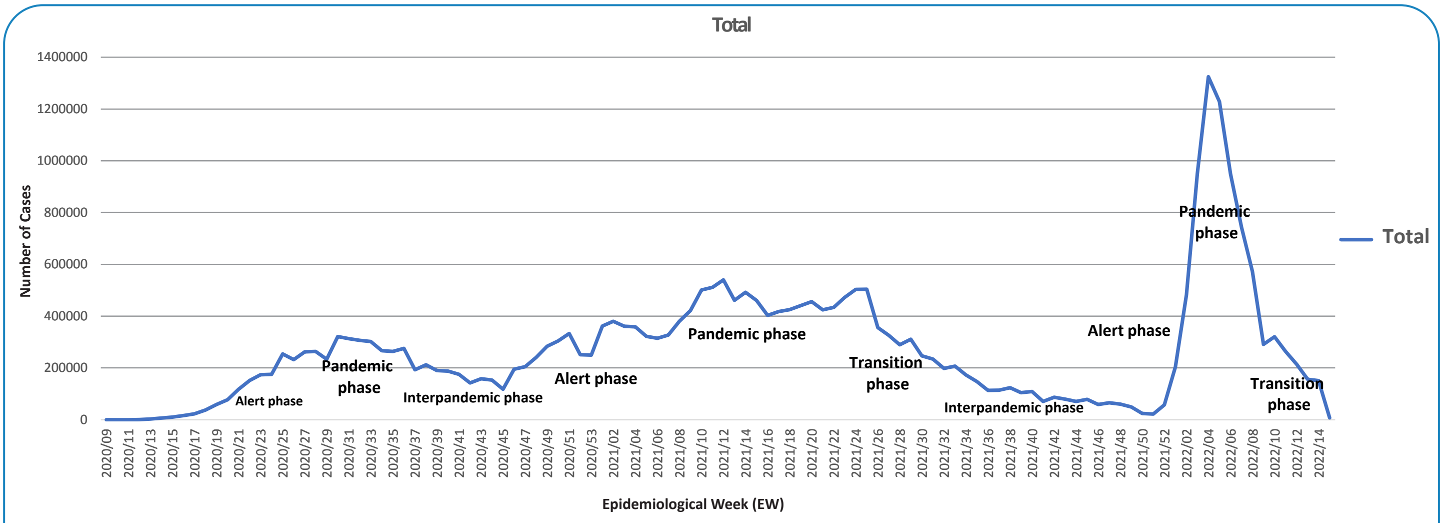

**FIGURE 1B:** The continuum of the phases: COVID-19.
**Source:** Authors.

in cases of other variants of concern; this may require a greater immune response from the vaccines and the prior infection itself. The asymptomatic transmission of the virus and the inability of infection or vaccination to provide durable protection against re-infection, make disease control strategies difficult[10-15].

Thus, vaccination is unlikely to completely stop the spread of SARS-CoV-2; however, even without reaching the threshold of herd immunity, studies have shown that vaccination reduces hospitalizations and deaths from COVID-19[11,12].

While science and scientific evidence are contributing to a better understanding of the virus and the disease, some operational and health service organization criteria need to be implemented as follows:1) Effective drugs, already authorized by other international agencies for the treatment of vulnerable patients, needs to be available at the Unified Health System (*Sistema Único de Saúde*). 2) Clinical care protocols should be implemented at all levels of health services: maintaining and strengthening surveillance of SARS-CoV-2 at all health levels is necessary to track the spread and evolution of the virus. This is important to detect and characterize new variants. Maintaining more intensive surveillance is vital to minimize transmission and morbidity, especially among the most vulnerable. 3) Effective campaigns on the need for biannual or annual booster doses are required, and the strategies adopted by immunization programs should be prioritized to minimize deaths, serious illness, and the overall burden of disease, and the impact on health systems, as well as reducing the future risks of emerging new variants. Booster vaccination is needed to maintain a high level of effectiveness against SARS-CoV-2. 4) Implementation of services to care for the sequelae of COVID-19 are required; this includes improved detection of the post-COVID-19 condition (Long COVID) which will be necessary to reduce long-term morbidity even after the pandemic has ended. 5) Periodic evaluations every 90 days, as adopted in developed countries should be conducted. 6) The capacity of field epidemiology and laboratory teams need to be strengthened to improve etiologic investigation. 7) Implementation of an acute respiratory syndrome surveillance strategy, integrating COVID-19 into the differential diagnosis portfolio of virus genomic surveillance, is required. It is important to emphasize the importance of information systems, such as the Influenza Epidemiological Surveillance System (*Sistema de Vigilância Epidemiológica da Gripe*) for severe cases, as well as the need to expand the representativeness of sentinel surveillance of influenza syndromes by region; For example, increasing sampling from 5 to 10 samples per collection unit and standardizing the virus panel in the Central Laboratories of Public Health of the States (*Laboratório Central de Saúde Pública*). Both representative and targeted sequencing are important for monitoring virus evolution and detecting new variants that may pose a threat to public health. 8) The capacity to investigate suspicious deaths should be expanded, and the performance of autopsies should be encouraged[16].

Tackling COVID-19 in just two years allows us to review lessons learned, re-evaluate approaches, and develop a framework that allows municipalities, states, the Federal District, and the Union to accelerate progress to control and prepare for the next emergencies. From the health care unit perspective, the system must be able to detect, notify, and respond in a timely manner to any change in epidemiological patterns[17]. At this stage of the pandemic, it is appropriate to discuss and agree on objective parameters for changing actions at the administrative level (operational concepts), based on scientific evidence

Finally, the closure of the Public Health Emergency of National Importance (*Emergência de Saúde Pública de Importância Nacional*) can and should be discussed, not with the intention of adopting unilateral and imposing measures, but so that the legacies of the pandemic can be incorporated. So that we are prepared to maintain the emergency status or even, if the emergency is eventually closed, we can rationally agree on a new declaration if the virus so imposes.

OPEN ACCESS


REFERENCES

1 Centers for Disease Control and Prevention (CDC) Principles of Epidemiology in Public Health Practice 3rd ed Atlanta CDC 2012 511p https://www.cdc.gov/csels/dsepd/ss1978/lesson1/section11.html
2 Medronho R Carvalho DM Bloch KV Luiz RR Werneck GL Epidemiologia 2 th ed São Paulo Atheneu 2009 685p
3 Bonita R Beaglehole R Kjellström T Basic Epidemiology 2nd. ed Geneva WHO 2006 230 p https://apps.who.int/iris/handle/10665/43541
4 World Health Organization (WHO) WHO Director-General’s opening remarks at the media briefing on COVID-19 11 03 2020 Geneva WHO https://www.who.int/director-general/speeches/detail/who-director-general-s-opening-remarks-at-the-media-briefing-on-covid-19---11-march-2020
5 Brasil. Agência Nacional de Vigilância Sanitária (Anvisa) Regulamento Sanitário Internacional RSI-2005 Brasília Anvisa 2009 79 p
6 World Health Organization (WHO) WHO Director-General's statement on IHR Emergency Committee on Novel Coronavirus (2019-nCoV) . 30 01 2020 Geneva WHO https://www.who.int/director-general/speeches/detail/who-director-general-s-statement-on-ihr-emergency-committee-on-novel-coronavirus-(2019-ncov)
7 Brasil Portaria n° 7.616, de 17 de novembro de 2011. Dispõe sobre a declaração de Emergência em Saúde Pública de Importância Nacional-ESPIN e institui a Força Nacional do Sistema Único de Saúde-FN-SUS Brasília Casa Civil 2011
8 Brasil. Mistério da Saúde (MS) Boletim epidemiológico nº 107- Boletim COE coronavírus Brasília MS 2022 https://www.gov.br/saude/pt-br/centrais-de-conteudo/publicacoes/boletins/boletins-epidemiologicos/covid-19/2022/boletim-epidemiologico-no-107-boletim-coe-coronavirus.pdf/view
9 World Health Organization (WHO) Pandemic influenza risk management: a WHO guide to inform & harmonize national & international pandemic preparedness and response Geneva World Health Organization (WHO) 2017 62 p https://apps.who.int/iris/handle/10665/259893
10 Antia R Halloran ME Transition to endemicity: Understanding COVID-19 Immunity 2021 54 10 2172 2176 S1074761321004040 34626549 10.1016/j.immuni.2021.09.019 PMC8461290
11 Siqueira PC Cola JP Comerio T Sales CMM Maciel EL Herd immunity threshold for SARS-CoV-2 and vaccination effectiveness in Brazil J Bras Pneumol 2022 48 2 e20210401 10.36416/1806-3756/e20210401 PMC8836627 35649044
12 Fine P Eames K Heymann DL ‘‘Herd Immunity’’: A Rough Guide Clinical Infectious Diseases 2011 52 7 911 916 https://academic.oup.com/cid/article/5 21427399 10.1093/cid/cir007
1 Cerqueira-Silva T Andrews JR Boaventura VS Ranzani OT Oliveira VDA Paixão ES Effectiveness of CoronaVac, ChAdOx1 nCoV-19, BNT162b2, and Ad26.COV2.S among individuals with previous SARS-CoV-2 infection in Brazil: a test-negative, case-control study Lancet Infect Des 2022 S1473-3099 22 00140 00142 https://www.thelancet.com/journals/laninf/article/PIIS1473-3099(22)00140-2/fulltext 10.1016/S1473-3099(22)00140-2 PMC8971277 35366959
14 World Health Organization (WHO) Tracking SARS-CoV-2-variants Geneva WHO 2021 https://www.who.int/en/activities/tracking-SARS-CoV-2-variants/
15 Morens DM Folkers GK Fauci AS The Concept of Classical Herd Immunity May Not Apply to COVID-19 J Infect Dis 2022 jiac109 10.1093/infdis/jiac109 PMC9129114 35356987
16 World Health Organization (WHO) Strategic Preparedness, Readiness and Response Plan to End the Global COVID-19 Emergency in 2022 Geneva WHO 2022 23 p https://www.who.int/publications/i/item/WHO-WHE-SPP-2022.1
17 Frieden TR Lee CT Bochner AF Buissonnière M McClelland A 7-1-7: an organising principle, target, and accountability metric to make the world safer from pandemics Lancet 2021 398 10300 638 640 https://www.thelancet.com/journals/lancet/article/PIIS0140-6736(21)01250-2/fulltext 34242563 10.1016/S0140-6736(21)01250-2 PMC9636000
